# LncRNAs down-regulate Myh1, Casr, and Mis18a expression in the Substantia Nigra of aged male rats

**DOI:** 10.18632/aging.102321

**Published:** 2019-10-02

**Authors:** Guoliang Zhang, Yunxiao Kang, Xu Feng, Rui Cui, Qiqing Guo, Xiaoming Ji, Yuanxiang Huang, Yannan Ma, Shufeng Liu, Geming Shi

**Affiliations:** 1 Department of Neurobiology, Hebei Medical University, Hebei Province, Shijiazhuang 050017, China; 2 Department of Human Anatomy, Hebei Medical University, Hebei Province, Shijiazhuang 050017, China; 3 Hebei Laboratory Animal Center, Hebei Medical University, Hebei Province, Shijiazhuang 050017, China; 4 Grade 2015 Eight-year Clinical Medicine Program, School of Basic Medical Sciences, Hebei Medical University, Hebei Province, Shijiazhuang 050017, China; 5 Neuroscience Research Center, Hebei Medical University, Hebei Province, Shijiazhuang 050017, China; 6 Hebei Key Laboratory of Forensic Medicine, Department of Forensic Medicine, Hebei Medical University, Hebei Province, Shijiazhuang 050017, China

**Keywords:** Substantia Nigra, messenger RNAs, long non-coding RNAs, circular RNAs, aged male rats

## Abstract

In this study, we used high-throughput RNA sequencing to identify mRNAs, long non-coding RNAs (lncRNAs) and circular RNAs (circRNAs) that are differentially expressed in the Substantia Nigra (SN) of aged and young rats. Gene Ontology and Kyoto Encyclopedia of Genes and Genomes pathway analyses were used to perform functional annotation of mRNAs that were either differentially expressed themselves (DEMs), targeted by differentially expressed lncRNAs (DELs), or the parents of differentially expressed circRNAs (DECs). A total of 112 DEMs, 163 DELs, and 98 DECs were found in the SN of aged rats. The down-regulated lncRNA NONRATT010417.2 targeted the down-regulated mRNA Myh1, while the down-regulated lncRNA NONRATT015586.2 and the up-regulated lncRNAs NONRATT000490.2 and NONRATT007029.2 all targeted the down-regulated mRNAs Casr and Mis18a. Western blots and RT-qPCR revealed that Myh1, Casr, and Mis18a protein and mRNA expression were significantly reduced in aged rats compared to young rats. This study improves our understanding of the transcriptional alterations underlying aging-related changes in the SN and provides a foundation for future studies of associated molecular mechanisms.

## INTRODUCTION

Aging is associated with the accumulation of deficits in cognition and motor control and with increased risk of neurodegenerative diseases, such as Parkinson’s disease [1–3]. The nigrostriatal dopaminergic pathway consists of projections from dopaminergic cell bodies in the Substantia Nigra (SN) to the corpus striatum, including the caudate, putamen, and globus pallidus [4]. The dopaminergic neurons of the SN are vulnerable to age-related degenerative processes [5]. In normal aging, the number and dopamine content of dopaminergic neurons in the SN progressively decreases, which leads to aging-related behavioral deficits [6, 7]. The dopaminergic neurons of the SN are vulnerable to oxidative stress [8] and inflammatory attack [9]. Reduced dopamine activity during normal aging is also linked to age-related mitochondrial DNA damage [10]. The mechanisms responsible for dysfunction and degeneration of the SN dopamine neurons in normal aging are complex and are not yet fully understood.

Age-related transcriptome changes may contribute to physiological changes in the dopaminergic neurons of the SN during aging. High-throughput RNA sequencing technologies have helped demonstrate that less than 3% of human transcripts are protein-coding RNAs; the rest are non-protein-coding RNAs, including long non-coding RNAs (lncRNAs) and circular RNAs (circRNAs) [11, 12]. Changes in protein expression contribute to changes in cellular function that characterize the aging process. It is therefore necessary to examine the expression of age-associated genes. Increasing evidence indicates that lncRNAs and circRNAs play crucial roles in various biological processes and diseases [13–19]. LncRNAs modulate gene expression via transcriptional, post-transcriptional, translational, and post-translational regulation [15, 20]. CircRNAs can also play important roles during and after transcription by functioning as microRNA (miRNA) sponges, regulating splicing, and interacting with RNA-binding proteins [21–23]. It is therefore important to examine changes in lncRNAs and circRNAs in addition to messenger RNAs (mRNAs) in the SN during normal aging.

In this study, we used high-throughput RNA sequencing to investigate mRNA, lncRNA, and circRNAs expression profiles. We identified RNAs that are differentially expressed in the SNs of 6-month-old rats and 24-month-old rats. We then identified Gene Ontology (GO) terms and Kyoto Encyclopedia of Genes and Genomes (KEGG) pathways associated with the differentially expressed mRNAs (DEMs), the mRNAs targeted by differentially expressed lncRNAs (DELs), and the parental mRNAs of the differentially expressed circRNAs (DECs). Finally, we evaluated the functional annotations of the DEMs targeted by DELs or that were parents of DECs to identify putative regulatory roles using bioinformatics approaches. Our findings help elucidate the molecular mechanisms by which mRNAs, lncRNAs, and circRNAs contribute to the cognitive and motor impairments as well as the functional deficits observed in SN dopaminergic neurons that occur during normal aging.

## RESULTS

### Age-dependent DEMs, DELs, and DECs in the SN

**Figure 1:**
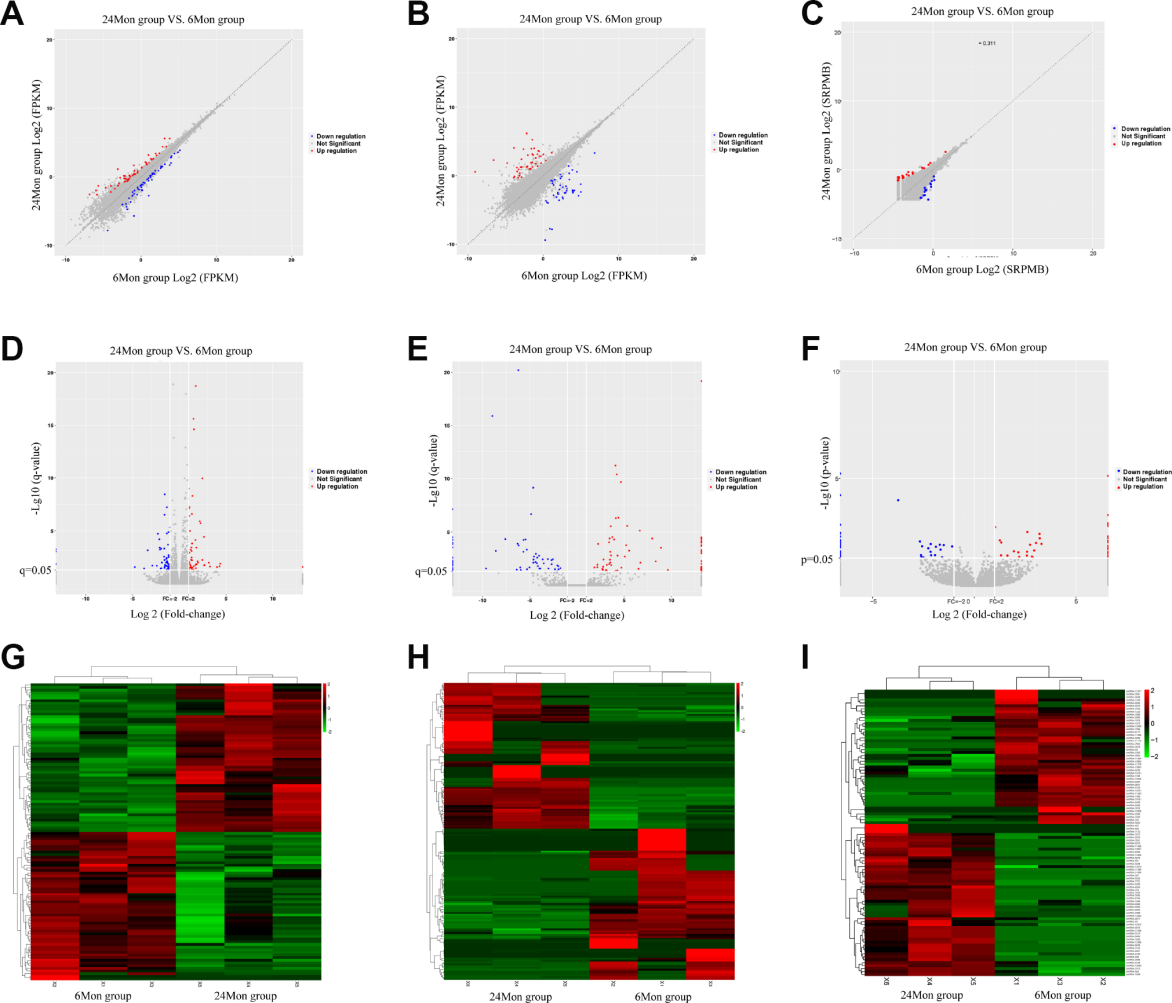
Scatter diagrams (**A**–**C**), volcano diagrams (**D**–**F**), and hierarchical clustering analysis (**G**–**I**) of relative mRNA (**A**, **D**, **G**), lncRNA (**B**, **E**, **H**), and circRNA (**C**, **F**, **I**) expression signals in the SN of 6Mon and 24Mon rats.

A total of 112 DEMs (Supplementary Table 1), 163 DELs (Supplementary Table 2), and 98 DECs (Supplementary Table 3) were found in the SN of 24-month old (24Mon) rats comparted to six-month old (6Mon) rats. Of these, 56 DEMs (50.0% of all DEMs), 80 DELs (49.1% of all DELs), and 52 DECs (53.1% of all DECs) were up-regulated, while 56 DEMs (50.0%), 83 DELs (50.9%), and 46 DECs (46.9%) were down-regulated, in the SN of 24Mon rats. Scatter diagrams (A-C), volcano diagrams (D-F), and hierarchical clustering analysis (G-I) showing differences in SN mRNA, lncRNA, and circRNA expression between 24Mon and 6Mon rats are shown in Figure 1. The ten DEMs for which expression changed the most in the SN of 24Mon rats compared to 6Mon rats are listed in Table 1.

**Table 1:** Top 10 up- and down-regulated DEMs in the SN of 24Mon rats.

**Gene id**	**Gene name**	**Description**	**Fold change**	**P-value**	**Regulation**
ENSRNOG00000047706	RT1-CE16	RT1 class I, locus CE16	Inf	2.95E-06	DOWN
ENSRNOG00000050134	AABR07042599.1	Uncharacterized protein	Inf	5.27E-06	DOWN
ENSRNOG00000001865	Serpind1	serpin peptidase inhibitor, clade D (heparin cofactor), member 1	Inf	0.00018	DOWN
ENSRNOG00000021555	Mis18a	MIS18 kinetochore protein A	-4.75492	0.000454	DOWN
ENSRNOG00000060523	Rps2-ps2	ribosomal protein S2, pseudogene 2	-3.75752	0.000683	DOWN
ENSRNOG00000049695	Myh1	myosin, heavy chain 1, skeletal muscle, adult	-3.37537	3.84E-06	DOWN
ENSRNOG00000020953	Ms4a7	membrane-spanning 4-domains, subfamily A, member 7	-2.88917	0.000289	DOWN
ENSRNOG00000025946	Igf2bp2	insulin-like growth factor 2 mRNA binding protein 2	-2.40051	0.000306	DOWN
ENSRNOG00000002265	Casr	calcium-sensing receptor	-2.28839	6.04E-08	DOWN
ENSRNOG00000013426	Mrgprf	MAS-related GPR, member F	-2.13363	2.38E-07	DOWN
ENSRNOG00000037167	Rtp3	receptor (chemosensory) transporter protein 3	Inf	0.000422	UP
ENSRNOG00000020400	Mef2b	myocyte enhancer factor 2B	4.379482	0.000155	UP
ENSRNOG00000050325	Emr4	EGF-like module containing, mucin-like, hormone receptor-like sequence 4	4.295064	0.000351	UP
ENSRNOG00000054834	AABR07001734.1	-	4.220192	2.08E-32	UP
ENSRNOG00000002776	Sell	selectin L	4.029786	0.000445	UP
ENSRNOG00000002031	Naa11	N(alpha)-acetyltransferase 11, NatA catalytic subunit	3.278133	0.000379	UP
ENSRNOG00000042220	Vsx1	visual system homeobox 1	3.162037	0.000119	UP
ENSRNOG00000007664	Tnfrsf13c	tumor necrosis factor receptor superfamily, member 13c	2.702566	0.000239	UP
ENSRNOG00000032246	Acsm3	acyl-CoA synthetase medium-chain family member 3	2.653876	0.000266	UP
ENSRNOG00000000451	RT1-Ba	RT1 class II, locus Ba	2.639609	1.30E-07	UP

**Figure 2:**
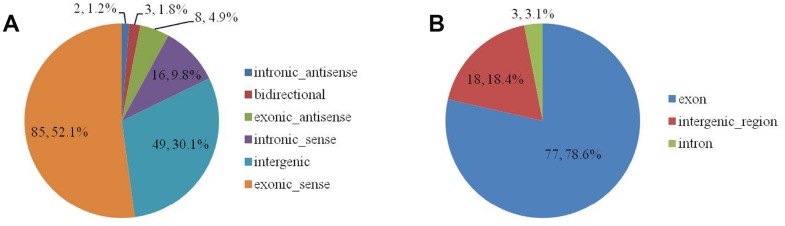
The biotype of DELs (**A**) and DECs (**B**) in the SN of 6Mon and 24Mon rats.

The 163 DELs were divided among six biotype categories: three (1.8%) were bidirectional, eight (4.9%) were exonic-antisense, 85 (52.1%) were exonic-sense, 49 (30.1%) were intergenic, two (1.2%) were intronic-antisense, and 16 (9.8%) were intronic-sense (Supplementary Table 4 and Figure 2A). The 98 DECs were divided among three categories: 77 (78.6%) were exon, 18 (18.4%) were intergenic-region, and three (3.1%) were intron (Supplementary Table 5 and Figure 2B).

### Functional annotation of DEMs

**Figure 3:**
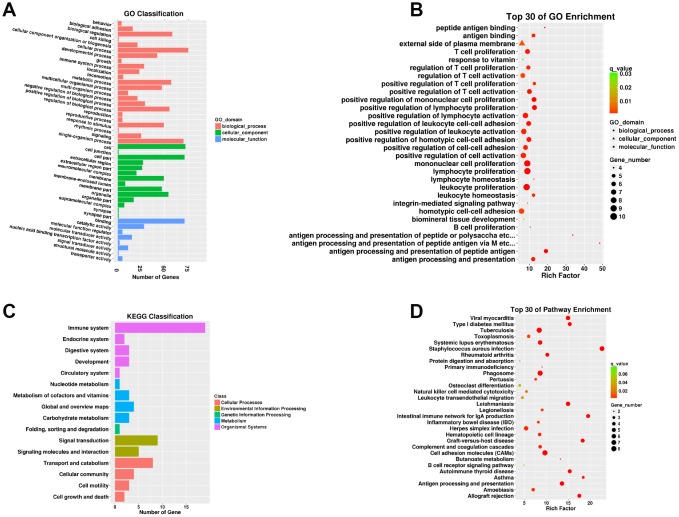
**Functional annotation of DEMs in the SN of 6Mon and 24Mon rats.** (**A**) GO classification, (**B**) Top 30 GO enrichments, (**C**) KEGG classifications, (**D**) Top 30 KEGG pathway enrichments.

A total of 112 DEMs were obviously responsive to 23 biological processes (BP), 14 cellular components (CC), and eight molecular functions (MF) in the SN of 24Mon rats (Figure 3A and Supplementary Table 6). These DEMs were associated with 290 functional enrichments (Supplementary Table 7). The top 30 GO enrichments are shown in Figure 3B. KEGG classifications indicated that these DEMs were responsive to four cellular processes and to two environmental information processing, one genetic information processing, four metabolism, and five organismal systems (Figure 3C and Supplementary Table 8). The DEMs were significantly enriched in 77 KEGG pathways (Supplementary Table 9). The top 30 KEGG pathway enrichments are shown in Figure 3D.

### Functional annotation of mRNAs targeted by DELs

**Figure 4:**
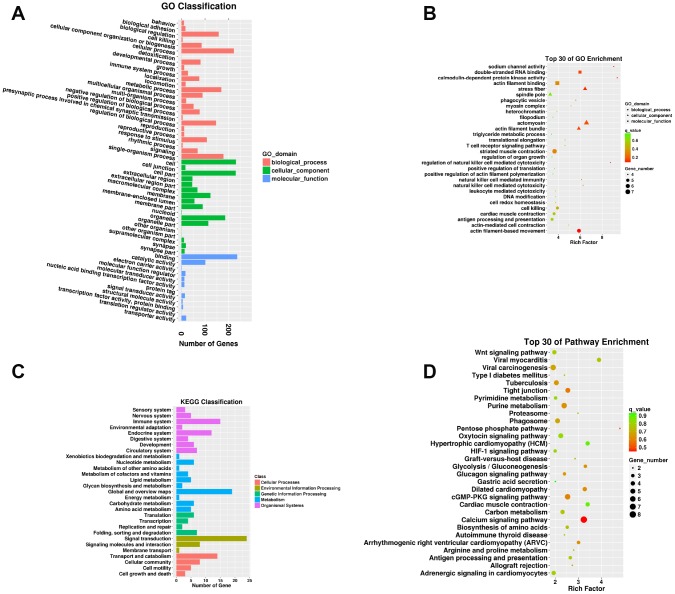
**Functional annotation of mRNAs targeted by DELs in the SN of 6Mon and 24Mon rats.** (**A**) GO classification, (**B**) Top 30 GO enrichments, (**C**) KEGG classifications, (**D**) Top 30 KEGG pathway enrichments.

A total of 737 targeting relationships were identified between DELs and mRNAs (Supplementary Table 10). The mRNAs targeted by DELs were obviously responsive to 25 BPs, 17 CCs, and 12 MFs in the SN of 24Mon rats (Figure 4A and Supplementary Table 11) and were associated with 858 functional enrichments (Supplementary Table 12). The top 30 GO enrichments are shown in Figure 4B. KEGG classifications indicated that the mRNAs targeted by DELs were responsive to four cellular processes and to three environmental information processing, four genetic information processing, ten metabolism, and eight organismal systems (Figure 4C and Supplementary Table 13). The mRNAs targeted by DELs were significantly enriched in 170 KEGG pathways (Supplementary Table 14). The top 30 KEGG pathway enrichments are shown in Figure 4D.

### Functional annotation of mRNAs that were parents of DECs

**Figure 5:**
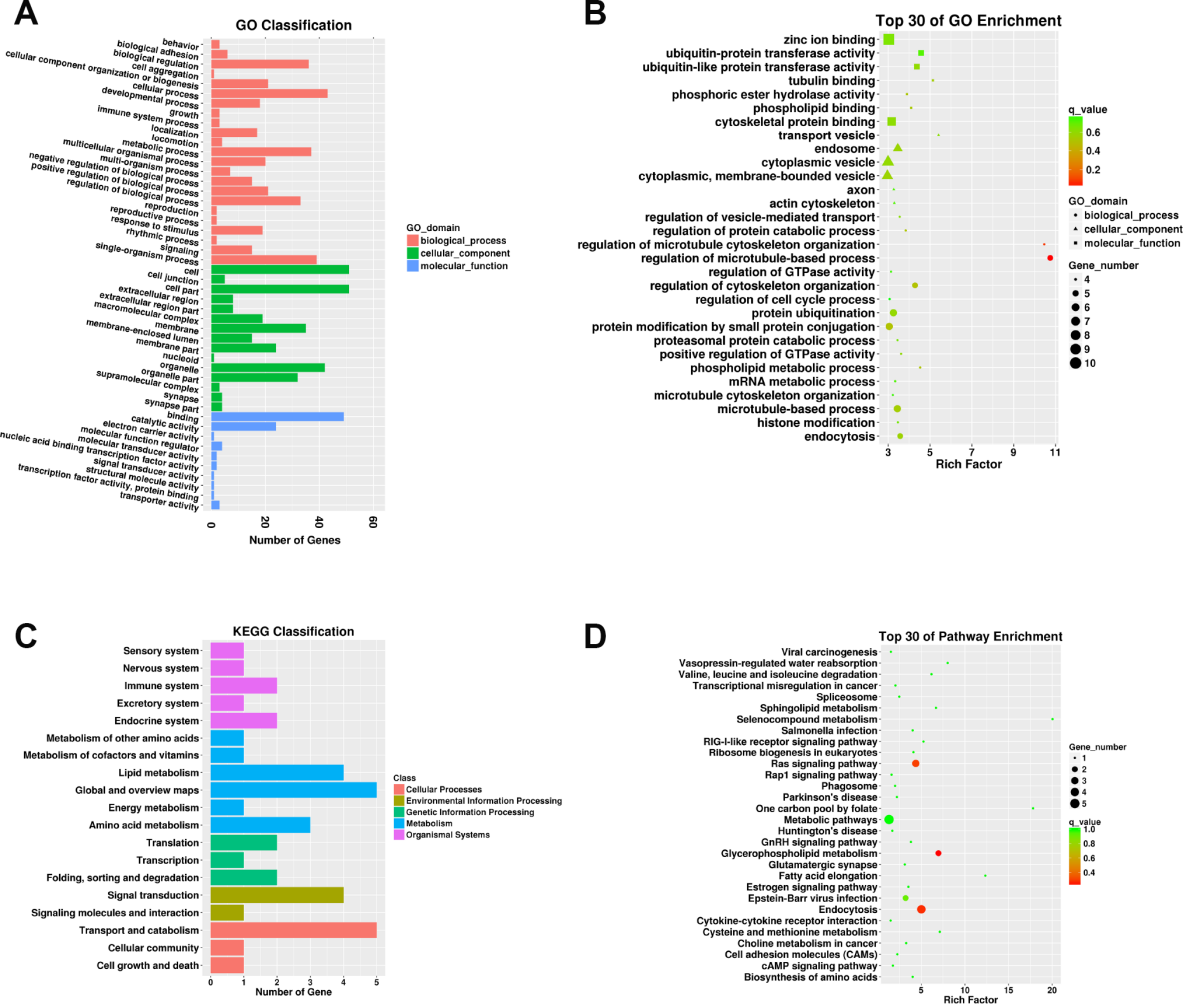
**Functional annotation of parental mRNAs of DECs in the SN of 6Mon and 24Mon rats.** (**A**) GO classification, (**B**) Top 30 GO enrichments, (**C**) KEGG classifications, (**D**) Top 30 KEGG pathway enrichments.

A total of 67 parental relationships were identified between DECs and mRNAs (Supplementary Table 15). The parental mRNAs of DECs were obviously responsive to 21 BPs, 15 CCs, and ten MFs in the SN of 24Mon rats (Figure 5A and Supplementary Table 16) and were associated with 258 functional enrichments (Supplementary Table 17). The top 30 GO enrichments are shown in Figure 5B. KEGG classifications indicate that these parental mRNAs are responsive to three cellular processes and to two environmental information processing, three genetic information processing, six metabolism, and five organismal systems (Figure 5C and Supplementary Table 18). The parental mRNAs were significantly enriched in 60 KEGG pathways (Supplementary Table 19). The top 30 KEGG pathway enrichments are shown in Figure 5D.

### Analysis of DEMs targeted by DELs and DEMs that are parents of DECs

**Figure 6:**
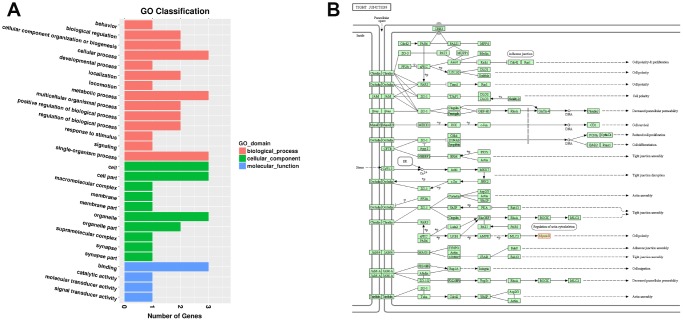
**Functional annotation of DEMs targeted by DELs in the SN of 6Mon and 24Mon rats.** (**A**) GO classification, (**B**) KEGG pathway analysis showed that Myh1 was responsive to cellular processes and significantly enriched in the tight junction (rno04530).

Among the 163 DELs, four (NONRATT010417.2, NONRATT015586.2, NONRATT000490.2, and NONRATT007029.2) targeted three DEMs (Myh1, Casr, and Mis18a) (Table 2). None of the parental mRNAs of the 98 DECs were differentially expressed. In the SN of 24Mon rats, down-regulated lncRNA NONRATT010417.2 targeted the down-regulated mRNA Myh1 (ENSRNOG00000049695), while the down-regulated lncRNA NONRATT015586.2 and the up-regulated lncRNAs NONRATT000490.2 and NONRATT007029.2 targeted the down-regulated mRNAs Casr (ENSRNOG00000002265) and Mis18a (ENSRNOG00000021555) (Table 2). GO analysis indicated that these three DEMs (Myh1, Casr, and Mis18a) were obviously responsive to 14 BPs, ten CCs, and four MFs in the SN of 24Mon rats (Figure 6A and Supplementary Table 20) and were not significantly associated with any GO functional enrichments. KEGG pathway analysis revealed that Myh1 is responsive to cellular processes and significantly enriched in the tight junction (rno04530). Myh1 was related to cell polarity (Figure 6B). Casr and Mis18a were neither responsive to any categories nor enriched in any pathways in the KEGG analysis.

**Table 2:** DELs target DEMs in the SN of 24Mon rats.

**LncRNA_id**	**Fold change**	**Pvalue**	**Qvalue**	**Regulation**	**Target gene_id**	**Target gene name**	**Fold change**	**Pvalue**	**Qvalue**	**Target gene Regulation**
NONRATT010417.2	-4.54774	5.26E-06	0.002014	DOWN	ENSRNOG00000049695	Myh1	-3.37537	3.84E-06	0.000685	DOWN
NONRATT000490.2	7.972086	4.56E-08	4.10E-05	UP	ENSRNOG00000002265	Casr	-2.28839	6.04E-08	1.90E-05	DOWN
NONRATT000490.2	7.972086	4.56E-08	4.10E-05	UP	ENSRNOG00000021555	Mis18a	-4.75492	0.000454	0.026922	DOWN
NONRATT007029.2	2.248629	2.30E-07	0.000131	UP	ENSRNOG00000002265	Casr	-2.28839	6.04E-08	1.90E-05	DOWN
NONRATT007029.2	2.248629	2.30E-07	0.000131	UP	ENSRNOG00000021555	Mis18a	-4.75492	0.000454	0.026922	DOWN
NONRATT015586.2	-1.74659	0.000117	0.024454	DOWN	ENSRNOG00000002265	Casr	-2.28839	6.04E-08	1.90E-05	DOWN
NONRATT015586.2	-1.74659	0.000117	0.024454	DOWN	ENSRNOG00000021555	Mis18a	-4.75492	0.000454	0.026922	DOWN

### Myh1, Casr, and Mis18a protein and mRNA expression in the SN of 6Mon and 24Mon rats

**Figure 7:**
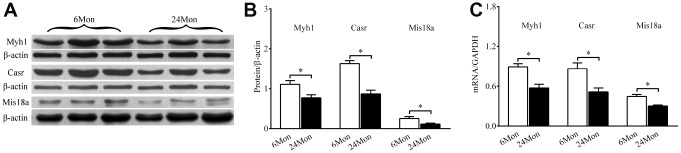
**Western blot and RT-qPCR showing Myh1, Casr, and Miss18a expression in the SN of 6Mon and 24Mon rats.** (**A**) Western blot images, (**B**) Bar graphs illustrating Myh1, Casr, and Mis18a protein expression, (**C**) Bar graphs illustrating Myh1, Casr, and Mis18a mRNA expression, *P < 0.01.

Western blot analysis was used to measure levels of Myh1, Casr, and Mis18a proteins extracted from the SN (Figure 7A). Expression of all three proteins was significantly reduced in 24Mon rats compared to 6Mon rats (Figure 7B). RT-qPCR revealed that Myh1, Casr, and Mis18a mRNA expression were significantly reduced in 24Mon rats compared to 6Mon rats (Figure 7C).

## DISCUSSION

In this study, we identified a total of 112 mRNAs that are differentially expressed in the SNs of 24- and six-month old rats. Of these DEMs, 56 were down-regulated and 56 were up-regulated in the SN of aged rats. GO analysis indicated that the DEMs were obviously responsive to 23 BPs, 14 CCs, and eight MFs and were associated with 290 functional enrichments. KEGG pathway analysis revealed that the DEMs were responsive to four cellular processes and to two environmental information processing, one genetic information processing, four metabolism, and five organismal systems, and were significantly enriched in 77 KEGG pathways. Overall, our results suggest that a large number of genes and pathways could play pivotal roles in normal aging processes in the SN.

While the DEMs identified here may be particularly important for understanding aging-related dysfunction and degeneration of SN dopamine neurons, their roles in the SN are poorly understood. Compared to young rats, the ten most strongly down-regulated mRNAs in aged rats were RT1-CE16, AABR07042599.1, Serpind1, Mis18a, Rps2-ps2, Myh1, Ms4a7, Igf2bp2, Casr, and Mrgprf, and the most strongly up-regulated mRNAs were Rtp3, Mef2b, Emr4, AABR07001734.1, Sell, Naa11, Vsx1, Tnfrsf13c, Acsm3, and RT1-Ba (Table 1). Major histocompatibility complex (MHC) class I (known as RT1-CE in the rat) protein expression affects neuroinflammatory processes and immune-mediated neurodegeneration both in Parkinson’s disease and in general [24, 25]. Serpin peptidase inhibitor plays an important role in synapse development and regulates synaptic plasticity [26, 27]. Serpind1 is very weakly expressed in brain tissues [28]. MS4A family members (including Ms4a7) are likely involved in signal transduction in many different cell lineages [29]. Igf2bp2, which regulates oxidative phosphorylation, binds several mRNAs that encode mitochondrial respiratory chain complex subunits and interacts with complex I (NADH:ubiquinone oxidoreductase) proteins [30]. Igf2bp2 is strikingly enriched in developing axon tracts, including spinal commissural axons [31]. MEF2 family proteins are involved in cell differentiation, proliferation, migration, and apoptosis [32]. DNA methylation, which is an epigenetic mechanism commonly associated with gene silencing, regulates Naa11 [33]. Mrgprf is classified as a MAS-related G-protein coupled receptor, and its function is currently unknown [34]. The abnormal expression of these genes observed in this study might help illuminate new regulatory mechanisms in the aged SN.

Regulation of gene and protein expression by non-coding RNAs (ncRNAs) has become a popular research topic in recent years [35, 36]. NcRNAs, including lncRNAs and circRNAs, play an important role in brain biology and molecular pathologies associated with neurodegeneration [22, 37]. For example, lncRNAs and circRNAs affect cell proliferation, differentiation, quiescence, senescence, stress and immune response, and many other cellular functions associated with aging [12, 38–40]. LncRNAs can regulate gene expression in the following ways: inter-chromosomal interactions, formation of nuclear structures or R-loops, regulating post-transcriptional mRNA decay, regulating cellular localization of RNA- and DNA-binding proteins, acting as guides or decoys for transcription factors, providing scaffolds for chromatin modifying complexes, and acting as miRNA sponges [20]. Large numbers of circRNAs that are biosynthesized from transcripts expressed in neuronal cells have been identified in neuronal tissues [41]. CircRNAs can modulate miRNA function, regulate protein production and gene transcription, and act as miRNA sponges [22, 42]. The lncRNA HOTAIR affects differentiation of mesenchymal stem cells and is implicated in senescence-associated DNA-methylation [43]. The muscleblind-like-3 (MBNL3) splicing factor promotes hepatocellular carcinoma (HCC) by increasing paxillin (PXN) expression through the alternative splicing of lncRNA-PXN-AS1. In HCC tissues, overexpression of MBNL3 promotes the inclusion of exon 4 of lncRNA-PXN-AS1, shifts the PXN-AS1-S isoform to PXN-AS1-L, and upregulates PXN mRNA and protein expression [44]. The lncRNA uc.173 regulates growth of the intestinal mucosa and stimulates intestinal epithelial renewal by reducing levels of miRNA195 [45]. Down-regulation of lncRNA MALAT1 attenuates neuronal cell death by regulating miR-30a expression, which in turn suppresses Beclin1-dependent autophagy, in cerebral ischemic stroke [46]. The circRNA circAGFG1 exerts oncogenic effects by sponging miR-195-5p, which modulates cyclin E1 expression and promotes tumorigenesis and the development of triple-negative breast cancer [47]. Here, we identified 163 DELs and 98 DECs in the SN of 24Mon rats. A single DEL might target multiple mRNAs, and multiple DELs can also target the same mRNA (Supplementary Table 10). In addition, while each DEC is derived from a single parental mRNA, a single mRNA might produce multiple DECs (Supplementary Table 15). GO and KEGG pathway analysis was performed to investigate the biological functions of the genes targeted by DELs and the parental genes of DECs. Our results indicate that those genes are involved in many processes and pathways. Thus, the complicated changes in regulatory mechanisms that occur in the SN during aging require further investigation.

We also used bioinformatics approaches to predict the potential regulatory roles of DEMs targeted by DELs and DEMs that were parents of DECs. Seven target relationships between four DELs and three DEMs were identified in the aged SN. In aged rats, the down-regulated lncRNA NONRATT010417.2 targeted the down-regulated mRNA Myh1, while the down-regulated lncRNA NONRATT015586.2 and the up-regulated lncRNAs NONRATT000490.2 and NONRATT007029.2 targeted the down-regulated mRNAs Casr and Mis18a. Immunoblotting and RT-qPCR revealed that expression of the Myh1, Casr, and Mis18a proteins and mRNAs was also significantly reduced in aged male rats. In addition, GO analysis revealed that the DELs targeting Myh1, Casr, and Mis18a are involved in behavior (GO:0007610), binding (GO:0005488), developmental processes (GO:0032502), metabolic processes (GO:0008152), signal transducer activity (GO:0004871), molecular transducer activity (GO:0060089), and other classifications. KEGG pathway analysis indicated that Myh1 is responsive to cellular processes, significantly enriched in the tight junction (rno04530), and related to cell polarity. Myosins constitute a superfamily of ubiquitous, multifunctional, and structurally related motor proteins that catalyze the hydrolysis of ATP to power directed movement on filamentous actin. The myosin superfamily plays fundamental roles in neuronal function, plasticity, morphogenesis, and survival [48]. The non-muscle myosins are likely involved in neuronal motility and the extension and maintenance of axons and dendrites [49]. Casr is a widely expressed G-protein coupled receptor. In neurons, Casr responds to changes in calcium levels that are involved in neuronal growth and migration, synaptic plasticity, and a variety of other processes [50]. Down-regulation of Casr expression is associated with neuronal injury, calcium disturbances, increased production of reactive oxygen species, and decreased nitric oxide release [51]. Mis18a, which is down-regulated in the amygdala during exposure to low-novelty environments, was enriched for GO terms relating to chromatin and nucleosome assembly and remodeling [52]. Mis18a knockout causes severe chromosomal missegregation, inhibition of CENPA, and ultimately cell death [53]. Reduced Mis18a expression is associated with problems in the formation of the nuclear spindle and chromosome segregation [54]. Therefore, we hypothesized that low expression of Myh1, Casr, and Mis18a in the SN of aged male rats is likely regulated by lncRNA. However, the specific mechanism, such as the direct connection and regulation of the indicated lncRNAs with Myh1, Casr and Mis18a, remains unsolved in this study and should be investigated *in vitro* using cell culture model in future experiments.

Mitochondria play key roles in bioenergetics, generation of reactive oxygen species, apoptosis, signal transduction, calcium homeostasis, and cell survival and death [55, 56]. Mitochondrial dysfunction has emerged as a hallmark of the aging process and is linked to the development of numerous age-related pathologies, including neurodegenerative disorders [57–59]. Both nuclear genes and mitochondrial genes code for proteins that are involved in mitochondrial functions [60, 61]. Whether the expression of proteins related to mitochondrial function is also regulated by ncRNAs remains largely unknown. In our study, lncRNA NONRATT015586.2, which was down-regulated, and lncRNAs NONRATT000490.2 and NONRATT007029.2, which were up-regulated, all targeted the ATP synthase mitochondrial F1 complex assembly factor 1 (Atpaf1), mitochondrial ribosomal protein 63 (Mrp63), and mitochondrial ribosomal protein L51 (Mrpl51) mRNAs; there were no age-dependent differences in the expression of these mRNAs (Supplementary Table 10). Unfortunately, we were not able to examine expression of the Atpaf1, Mrp63, and Mrpl51 proteins. Studies have shown that abnormal expression of Atpaf1 and Mrpl51 is closely related to mitochondrial dysfunction [62, 63]. It is possible that differential expression of these mRNAs was not observed in this study due to the small number of samples examined (n=3) or because the identified lncRNAs affected translation rather than transcription.

In conclusion, we characterized mRNAs, lncRNAs, and circRNAs that were differentially expressed in the SN of aged and young rats. We found that the indicated lncRNAs down-regulated the expression of Myh1, Casr, and Mis18a in the aged rat SN. These findings help to elucidate transcriptional underpinnings of aging in the SN and provide a foundation for future studies on associated molecular mechanisms.

## MATERIALS AND METHODS

### Animals housing and ethics

A total of six male Wistar rats were assigned to either the 6Mon (n = 3) or the 24Mon (n = 3) group. All rats were supplied by the Experimental Animal Center of Hebei Medical University and were housed in an air-conditioned room (22 ± 2°C) on a 12-h light–dark cycle (lights on 06:00 h). Food and water were available *ad libitum*. All experimental procedures were conducted in accordance with the rules in the “Guidelines for the Care and Use of Mammals in Neuroscience and Behavioral Research” and were approved by the Committee of Ethics in Animal Experiments at Hebei Medical University.

### Sample preparation

All rats in each group were sacrificed by decapitation. The brains were removed quickly and the blocks containing the SN in the ventral midbrain (between 2.96 and 3.70 mm, Paxinos and Watson, 1998) was dissected on an ice-cold plate using a scalpel for ophthalmic surgery and a stereomicroscope. Tissue blocks containing the SN were processed for high-throughput RNA sequencing and western blot analysis.

### RNA extraction and purification

Total RNA was extracted from the SN using the RNeasy Mini Kit (Cat 74106, Qiagen) according the manufacturer’s instructions; RNA integrity number (RIN, from 0 to 10) was determined using an Agilent Bioanalyzer 2100 (Agilent Technologies, Santa Clara, CA, US). Higher RIN scores indicate greater RNA integrity; only qualified total RNA samples with RIN ≥ 7.0 were further purified using the RNAClean XP Kit (Cat A63987, Beckman Coulter, Inc., Kraemer Boulevard, Brea, CA, USA) and the RNase-Free DNase Set (Cat 79254, QIAGEN, GmBH, Germany).

### Sequencing library construction and sequencing

Purified SN RNA samples were used for library preparation. The TruSeq RNA Sample Preparation Kit (RS-122-2001, Illumina, San Francisco, CA, USA) was then used to synthesize paired-end libraries according to the manufacturer’s guidelines. Library construction, Illumina sequencing, sequence analysis of high-quality reads, and bioinformatic data analysis were performed by the Shanghai Biotechnology Corporation.

### RNA sequence mapping and bioinformatic analysis of differentially expressed mRNAs, lncRNAs, and circRNAs

Before read mapping, clean reads were isolated from the raw reads using Seqtk (https://github.com/lh3/seqtk). Hisat2 (version 2.0.4) was used for alignment of clean reads and genome mapping [64]. Relative expression levels of all matched unigenes were normalized by transforming the clean reads into fragment kilobase of exon per million fragments mapped (FPKM) for mRNAs and lncRNAs and spliced reads per billion mapping (SRPBM) for circRNAs. RNAs were considered significantly differentially expressed under the following criteria: (1) fold-change (FC) value > 2 (|log_2_FC|>1), and (2) adjusted false discovery rate (FDR), which was a q-value < 0.05 for mRNAs and lncRNAs and a p-value < 0.05 for circRNAs.

### GO and KEGG analysis

To better understand the biological functions and potential regulatory roles of dysregulated mRNAs, lncRNAs, and circRNAs, we performed GO (http://geneontology.org) and KEGG (https://www. kegg.jp) pathway and enrichment analysis. GO analysis involved classifying associations between DEMs, mRNAs targeted by DELs, and parental mRNAs of DECs and the following three categories: biological process (BP), cellular component (CC), and molecular function (MF). GO terms were widely used as functional enrichment analysis for many genes. KEGG pathway analysis included classifications and pathway enrichment analysis. A q-value < 0.05 denoted significant enrichments in the GO and KEGG pathways.

### Western blot analysis

The tissue blocks used for detection of Myh1, Casr, and Mis18a proteins were homogenized in radioimmunoprecipitation assay (RIPA) buffer containing 1% Triton X-100, 0.1% SDS, 0.5% sodium deoxycholate, and protease inhibitors (100 μg/mL phenylmethanesulfonyl fluoride, 30 μg/mL aprotinin, and 1 mM sodium orthovanadate), and then sonicated four times for 10 s each. After centrifugation at 12,000×g for 20 min at 4°C, the supernatant was collected and centrifuged again as described above. The resulting final supernatant was stored at -80°C until use. Samples from the SN (50 μg) were diluted with 2× sample buffer (50 mM Tris, pH 6.8, 2% SDS, 10% glycerol, 0.1% bromophenol blue, and 5% β-mercaptoethanol), heated for 5 min at 95°C before sodium dodecyl sulfate polyacrylamide gel electrophoresis (SDS-PAGE) on a 10% gel, and subsequently transferred to a polyvinylidene fluoride (PVDF) membrane (Millipore). The membrane was incubated for 2 h with 5% nonfat dry milk in Tris-buffered saline (TBS) containing 0.05% Tween 20 (TBST) (20 mM Tris-Cl, 137 mM NaCl, 0.1% Tween 20, pH 7.6) at room temperature. The membrane was rinsed in TBST three times and then incubated overnight with rabbit anti-Myh1 polyclonal antibody (Sigma, SAB2104768, 1:100), rabbit anti-Casr polyclonal antibody (Sigma, SAB4503369, 1:500), or rabbit anti-Mis18a polyclonal antibody (Biorbyt, orb325235, 1:200) at 4°C. After three washes, the membrane was incubated for 2 h in horseradish peroxidase-conjugated anti-rabbit IgG (1:6000). Immunoreactive bands were visualized using enhanced chemiluminescence (ECL, Amersham Biosciences) and film exposure (Kodak XBT-1). Following stripping, each PVDF membrane was subsequently immunoblotted with mouse anti-β-actin monoclonal antibody (Santa Cruz Biotechnology, Santa Cruz, CA, USA; sc-47778). The labeling densities for Myh1, Casr, and Mis18a were compared with those of β-actin, which served as the endogenous control. The specific bands were recorded by a scanner and analyzed by transmittance densitometry using Gel-Pro Analyzer Analysis software (Media Cybernetics).

### Real-time quantitative polymerase chain reaction (RT-PCR)

Total RNA (2 μg) from the SN was subjected to reverse transcription using random primer to obtain the first-strand cDNA template. Real-time fluorescence quantitative PCR was performed with 0.8-μl cDNA (diluted 1:10), 2-μl specific primers, and 2X GoTaq® Green Master Mix (Promega, USA) with a final volume of 20 μl. PCR was performed as follows: an initial cycle at 95 °C for 10min, followed by 40 cycles at 95 °C for 15 s, 58 °C for 20 s, and 72 °C for 27 s. The products were then analyzed by melting curve to confirm the specificity of amplification. Expression of Myh1, Casr and Mis18a genes was analyzed using glyceraldehyde-3-phosphate dehydrogenase (GAPDH) as an internal control. The sets of primers were as follows: Myh1 (5′-CCTGGATGA TCTACACCTACTC-3′ and 5′-GTCAGAGATAGAGA AGATGTGGG-3′), Casr (5′-TGACGAGCCTCAGAAG AATGC-3′ and 5′-CCTCCACCACTAATGACGAAGC-3), Mis18a (5′-TGGAACCCTGCTCTACCTCT-3′ and 5′-CCAATGGCGGCTCGAATCTT-3′), GAPDH (5′-TG AACGGGAAGCTCACTG-3′ and 5′-GCTTCACCAC CTTCTTGATG-3′).

### Statistical analysis

Data are reported as means ± SD. Independent-samples t-tests were used for statistical analysis. A value of p < 0.05 was considered significant.

## Supplementary Material

Supplementary Tables

Supplementary Table 1

Supplementary Table 2

Supplementary Table 3

Supplementary Table 4

Supplementary Table 5

Supplementary Table 6

Supplementary Table 7

Supplementary Table 8

Supplementary Table 9

Supplementary Table 10

Supplementary Table 11

Supplementary Table 12

Supplementary Table 13

Supplementary Table 14

Supplementary Table 15

Supplementary Table 16

Supplementary Table 17

Supplementary Table 18

Supplementary Table 19

Supplementary Table 20
