# Supplementary material for: LncRNAs down-regulate Myh1, Casr, and Mis18a expression in the Substantia Nigra of aged male rats
Source: Aging (Albany NY). 2019 Oct 2;11(19):8313–28. doi: 10.18632/aging.102321 (PMC6814601; doi:10.18632/aging.102321)
Supplement: Supplementary Tables [file aging-11-102321-s020.pdf]

SUPPLEMENTARY TABLES

Please browse Full Text version to see the data of Supplementary Table 1–20.

Supplementary Table 1. Differentially expressed mRNAs (DEMs).

Supplementary Table 2. Differentially expressed lncRNAs (DELs).

Supplementary Table 3. Differentially expressed circRNAs (DECs).

Supplementary Table 4. Biotype of DELs.

Supplementary Table 5. Biotype of DECs.

Supplementary Table 6. GO classification of DEMs.

Supplementary Table 7. GO enrichment of DEMs.

Supplementary Table 8. KEGG classification of DEMs.

Supplementary Table 9. KEGG enrichment of DEMs.

Supplementary Table 10. DELs target mRNAs.

Supplementary Table 11. GO classification of DELs target mRNAs.

Supplementary Table 12. GO enrichment of DELs target mRNAs.

Supplementary Table 13. KEGG classification of DELs target mRNAs.

Supplementary Table 14. KEGG enrichment of DELs target mRNAs.

Supplementary Table 15. DECs parental mRNAs.

Supplementary Table 16. GO classification of DECs parental mRNAs.

Supplementary Table 17. GO enrichment of DECs parental mRNAs.

Supplementary Table 18. KEGG classification of DECs parental mRNAs.

Supplementary Table 19. KEGG enrichment of DECs parental mRNAs.

Supplementary Table 20. GO classification of DELs target DEMs.
